# Empathy in Harmony: Music and The Person-Centered Approach in Encounter Groups

**DOI:** 10.1192/j.eurpsy.2026.11688

**Published:** 2026-07-31

**Authors:** K. Christopoulou, S. Georgakis, M. Gouva, M. Kefalopoulou

**Affiliations:** 1 Scientific Laboratory of Psychology & Person-Centered Care, University of Ioannina, Ioannina; 2 Department of Counselling, ICPS College, Athens, Greece

## Abstract

**Introduction:**

Empathy is a core condition of the Person-Centered Approach (PCA) and a central quality in therapeutic practice. Music, likewise, is a powerful medium for emotional resonance and connection. While prior studies suggest links between music, empathy, and perspective-taking, little is known about how structured person-centered encounter groups may foster empathy among musicians.

**Objectives:**

This study examined whether participation in encounter groups is associated with higher self-reported empathy among musicians, and whether sex differences emerge.

**Methods:**

Sixty-eight musicians (18–60 years) were divided into two groups: participants with no encounter group experience (n = 43) and those who attended eight PCA-based sessions (n = 25). All completed the Greek version of the Empathy Quotient (EQ). A two-way ANOVA tested the effects of group participation and sex on empathy scores.

**Results:**

Empathy scores ranged from 19 to 72 (M = 43.07, SD = 10.49). Encounter group participants and females showed slightly higher mean empathy levels than non-participants and males, though differences did not reach statistical significance.

**Conclusions:**

Preliminary findings suggest that person-centered encounter groups may hold potential for fostering empathy among musicians, aligning with the empathic foundations of the PCA. Although the observed differences were not significant, the trends point to the value of further research with larger samples and longer interventions. The study highlights music-based group experiences as a promising context for exploring empathy development, with implications for therapeutic, educational, and artistic settings.

**Disclosure of Interest:**

None Declared

